# Cell Signaling and Differential Protein Expression in Neuronal Differentiation of Bone Marrow Mesenchymal Stem Cells with Hypermethylated Salvador/Warts/Hippo (SWH) Pathway Genes

**DOI:** 10.1371/journal.pone.0145542

**Published:** 2015-12-29

**Authors:** Hui-Hung Tzeng, Chi-Hung Hsu, Ting-Hao Chung, Wen-Chien Lee, Chi-Hsien Lin, Wan-Chen Wang, Chen-Yu Hsiao, Yu-Wei Leu, Tzu-Hsien Wang

**Affiliations:** 1 Department of Chemical Engineering, Systems Biology and Tissue Engineering Research Center, National Chung Cheng University, Minhsiung, Chiayi, 621 Taiwan; 2 Department of Life Science, National Chung Cheng University, Minhsiung, Chiayi, 621, Taiwan; University of Dayton, UNITED STATES

## Abstract

Human mesenchymal stem cells (MSCs) modified by targeting DNA hypermethylation of genes in the Salvador/Warts/Hippo pathway were induced to differentiate into neuronal cells *in vitro*. The differentiated cells secreted a significant level of brain-derived neurotrophy factor (BDNF) and the expression of BDNF receptor tyrosine receptor kinase B (TrkB) correlated well with the secretion of BDNF. In the differentiating cells, CREB was active after the binding of growth factors to induce phosphorylation of ERK in the MAPK/ERK pathway. Downstream of phosphorylated CREB led to the functional maturation of differentiated cells and secretion of BDNF, which contributed to the sustained expression of pERK and pCREB. In summary, both PI3K/Akt and MAPK/ERK signaling pathways play important roles in the neuronal differentiation of MSCs. The main function of the PI3K/Akt pathway is to maintain cell survival during neural differentiation; whereas the role of the MAPK/ERK pathway is probably to promote the maturation of differentiated MSCs. Further, cellular levels of protein kinase C epsilon type (PKC-ε) and kinesin heavy chain (KIF5B) increased with time of induction, whereas the level of NME/NM23 nucleoside diphosphate kinase 1 (Nm23-H1) decreased during the time course of differentiation. The correlation between PKC-ε and TrkB suggested that there is cross-talk between PKC-ε and the PI3K/Akt signaling pathway.

## Introduction

Human mesenchymal stem cells (MSCs) derived from adult bone marrow can be cultured long-term, expanded in proper conditions, and show the ability to differentiate into neuronal cells *in vitro*. The induction of MSCs into neural cells *in vitro* was first attempted in 2000 by Woodbury et al. [1]. They used chemicals to induce mouse and human mesenchymal stem cells to differentiate into neurons. Firstly, the cells were cultured in β-mercaptoethanol (BME)-containing medium for 1 day and then in a differentiation-inducing medium containing dimethylsulfoxide (DMSO) and butylated hydroxyanisole (BHA). After 3 hours of induction, the cells showed the characteristics of neuronal cells, exhibiting neuron-specific enolase and neurofilament-M. In the same year, Sanchez-Ramos published results describing *in vitro* differentiation of human and rat mesenchymal stem cells into neuronal cells using growth factors EGF and BDNF. The differentiated cells expressed the precursor gene of neurons, nestin, and biomarkers of neuronal cells, including glial fibrillary acidic protein (GFAP) and neuron-specific nuclear protein (NeuN) [2]. Blondheim et al. found that human bone marrow MSCs exhibit a total of 12 neural genes, including Nestin, NSE, NeuN, and 11 nerve-related transcription factors, including neural zinc finger 3 (NZF-3) and paired box gene 3 (Pax3) [3]. Human MSCs also express eight dopamine neuron-related genes such as tyrosine hydroxylase (TH), aromatic l-amino acid decarboxylase (AADC), the transcription factor nuclear receptor related-1 (Nurr-1), and the paired-like homeodomain transcription factor-3 (Pitx-3). The literature confirms that MSCs have the ability to differentiate into neurons.

Since the discovery of the ability of MSCS to differentiate into neuronal cells, a variety of protocols for neuronal differentiation have been proposed. Although, neuronal differentiation after chemical stimulation for a few hours could be a misconception since the use of Triton, Tween-20, NaOH and NaCl can lead to similar results as induction using BME or DMSO/BHA [4]. Neuronal induction of MSCs into functional neuron-like cells has been generally achievable using cytokines. According to the literature, the cytokines used for neuronal induction can be classified as: (1) growth factors such as basic fibroblast growth factor (FGF-2), FGF-8, epidermal growth factor (EGF), nerve growth factor (NGF), and platelet-derived growth factor (PDGF); (2) substances that can raise the cellular concentration of cyclic adenosine monophosphate (cAMP) such as forskolin, 3-isobutyl-1-methylxanthine (IBMX), and dibutyryl cAMP; (3) neurotrophins such as NT-3, NT-5, BDNF, and glial cell line-derived neurotrophic factor (GDNF); and (4) other factors such as retinoic acid (RA). In addition to individual use, combinations of several classes of cytokines have also frequently been used for neuronal differentiation of MSCs [3, 5–12].

Among the predisposition genes reported by Blondheim et al. [3], Nurr-1 is the key transcription factor for the differentiation of human MSCs to dopamine neurons [13]. Induction of MSCs into dopamine-producing cells is of great interest because of their therapeutic potential in neurodegenerative disorders like Parkinson's disease [9–10, 14–15]. According to Trzaska et al., human bone marrow MSCs become dopaminergic neurons when incubated with a neuronal induction medium composed of SHH, bFGF, and FGF8 for 12 days [15]. A combination of SHH, FGF-8, and RA has also been used to produce dopaminergic-like neurons [14].

In the present study, isolated MSCs from bone marrow and *me*_SWH MSCs were differentiated into dopaminergic neurons using an induction medium containing SHH, bFGF, and FGF8. The *me*_SWH MSCs were obtained from human bone marrow MSCs by modification of genes in the Salvador/Warts/Hippo (SWH) pathway by targeting DNA hypermethylation. We subsequently examined changes in the proteomes of MSCs throughout neuronal differentiation. The signal transduction pathways involved in the regulation of dopaminergic differentiation of MSCs were also investigated. Differentially expressed proteins and signal proteins in MAPK/ERK and PI3K/Akt pathways were quantified by western blotting. We believe that understanding the molecular mechanisms regulating *in vitro* neuronal differentiation is crucial for the application of MSCs for cell therapy for the treatment of neurological disorders.

## Materials and Methods

### Neuronal induction of MSCs

Human MSCs isolated from bone marrow [16] were treated by the methylation of nine genes in the SWH signaling pathway to yield *me*_SWH-treated MSCs [17]. The *me*_SWH-treated MSCs retained markers of stemness, could be induced to differentiate, and were not shown to be tumorigenic in a nude mice assay. Both human MSCs and *me_*SWH-treated MSCs were cultured in a culture medium (CM) composed of Dulbecco’s Modified Eagle’s Medium (DMEM) (Sigma-Aldrich) and 10% fetal bovine serum (FBS) (Gibco). The medium was replaced with fresh CM every 3–4 days. When the cells reached 70–80% confluence, CM was removed and the cells were washed with phosphate-buffered saline (PBS) twice. Fresh neural induction medium (NIM) was then added. The NIM consisted of neurobasal medium (Gibco), 0.5% B-27 supplement (Gibco), 250 ng/ml SHH (Peprotech), 100 ng/ml FGF-8 (Peprotech), and 50 ng/ml bFGF (Peprotech), as previously described [15]. The medium was not changed during the neural induction period. For the study of signaling pathways regulating neuronal differentiation, TrkB inhibitor (0.5 μM), Akt inhibitor (10 μM), ERK inhibitor (10 μM), or BDNF (100 ng/ml) was also added to the induction medium. TrkB inhibitor (K-252a), Akt inhibitor IV in solution, and ERK inhibitor (PD98059) were obtained from Calbiochem.

### Determination of survival rate

MSCs were seeded on a 96-well plate with a cell density of 5000 cells/well. After neuronal induction, the number of viable proliferating cells was determined using the CellTiter 96® AQueous One Solution Cell Proliferation Assay (Promega). The assay was performed by adding 20 μl of the CellTiter 96® AQueous One Solution Reagent directly to culture wells, incubating in a 5% CO_2_ incubator at 37°C for 1–4 hours, and then recording absorbance at 490 nm in a 96-well plate reader. The CellTiter 96® AQ_ueous_ One Solution Reagent contains a tetrazolium compound MTS (3-(4,5-dimethylthiazol-2-yl)-5-(3-carboxymethoxyphenyl)-2-(4-sulfophenyl)-2H-tetrazolium, inner salt), which in the presence of phenazine methosulfate can be reduced by living cells to yield a colored formazan product. The quantity of formazan product as measured by absorbance at 490 nm is directly proportional to the number of living cells in culture.

### Determination of BDNF concentration by ELISA

The BDNF secreted by differentiated cells was detected using the *ChemiKine* ^TM^ BDNF Sandwich ELISA Kit obtained from Millipore. For each determination, the number of cells per milliliter was in the range of 1.1–3.7 ×10^5^.

### Two-dimensional gel electrophoresis (2-DE)

Before and after a 5-day differentiation induction, *me*_SWH-treated MSCs were harvested using trypsin-EDTA and centrifuged to yield cell pellets. These pellets were added to lysis buffer containing urea (7 M), thiourea (2 M), CHAPS (4% w/v), DTT (50 mM), and Bio-Lyte ampholyte, pH 3–10, (0.2%) for cell disruption under sonication for 30 min and then incubated for 1 h at room temperature. The resultant suspension was centrifuged at 20,000 × *g* at 4°C for 30 min and the supernatant was collected and then precipitated with a four-fold volume of acetone at -20°C for 16 h. The total protein content was quantified based on the method of Bradford. Protein precipitates were stored at -80°C prior to 2-DE.

2-DE was carried out using a previously published protocol [18–19]. Samples of protein precipitate (approx. 100 μg protein) were mixed with rehydration buffer containing urea (7 M), thiourea (2 M), CHAPS (2% w/v), DTT (50 mM), and Bio-Lyte ampholyte, pH 3–10, (0.2%), and the resultant mixture was applied to a 18 cm nonlinear pH 3–10 IPG strip (GE Healthcare) settled in the slot of a strip holder (BioRad) and rehydrated at 50 V for 16 h. Proteins underwent isoelectric focusing in a Protean IEF Cell (BioRad, Hercules, CA) programmed as follows: 500 V for 1 h; 1000 V for 1 h; 1000 to 8000 V within 2 h; 8000 V for 7 h; and finally 500 V for 12 h. The total voltage-hour for IEF was 65 kVh. After isoelectric focusing, strips were equilibrated in 2 sequential equilibrium buffers containing 2% (w/v) DTT and 2.5% (w/v) iodoacetamide for 15 min. Electrophoresis in the second dimension was carried out on a 12.5% SDS-polyacrylamide gel in a Protean II xi Cell (BioRad, Hercules, CA). Five replicates were performed in this study. Gels were then stained as previously described [20] with a modification consisting of the reduction of the concentration of silver nitrate to 0.2% (w/v). Stained gels were scanned on an ImageScanner densitometer (GE Healthcare, Fairfield, CT) at 300 dpi resolution with a blue filter and images were analyzed by ImageMaster 2D platinum software (version 5, GE Healthcare, Fairfield, CT). The expression level of each protein spot was quantified by the %vol, defined as the value of the intensity integration over the feature area of one spot divided by the total intensity integration over all of the spots in the whole gel image. The t-test was used for testing a difference in spot %vol between the two groups, before and after differentiation.

Protein spots of interest were sliced from silver-stained gels followed by in-gel digestion. The resultant digested peptide samples were subjected to MALDI MS and MS/MS analysis. For protein identification, MS results of peptide mass fingerprinting (PMF) and peptide fragment fingerprinting (PPF) were searched against the NCBI database using the Mascot program [19].

### Western blot assay

During the course of differentiation, MSC cultures were sampled for the assay of cellular signaling proteins. A western blot assay of ERK (ERK1+ERK2), phosphorylated ERK, TrkB, β-actin, and phosphorylated CREB was performed using the standard protocol. Developed films were scanned and quantitative analysis was performed using ImageJ software (National Institutes of Health, USA). The proportion of phosphorylated protein to total protein or the proportion of protein of interest to β-actin was normalized to the undifferentiated group. The antibodies mouse monoclonal to β-actin, rabbit polyclonal to TrkB, rabbit polyclonal to mouse, and goat polyclonal to rabbit were obtained from Abcam (Cambridge, MA, USA). Rabbit anti-CREB phosphospecific antibody, rabbit polyclonal anti-ERK1&2 phosphospecific antibody, and mouse anti-MAP kinase (ERK1+ERK2) antibody were purchased from Invitrogen (Carlsbad, CA, USA). Primary antibodies of ERK, phosphorylated ERK, TrkB, β-actin, and phosphorylated CREB were used at 1/500, 1/1500, 1/400, 1/5000, and 1/1500 dilutions, respectively. Two secondary antibodies, goat anti-rabbit IgG-HR and rabbit polyclonal to mouse IgG-HRP, were used at 1/5000 dilutions.

Differentially expressed proteins observed from 2-DE gels and identified by mass spectrometry were also assayed by western blotting. The housekeeping protein GAPDH was used as an internal control. The proportion of the protein of interest to GAPDH was normalized to the undifferentiated group. The primary antibodies rabbit monoclonal anti-PKC-ε and anti-GAPDH were obtained from Cell Signaling Technology, Inc. (Danvers, MA, USA); while rabbit polyclonal anti-UKHC and rabbit monoclonal anti-NM23-H1 were obtained from Santa Cruz Biotechnology, Inc. (Santa Cruz, CA, USA) and Abcam, respectively. Primary antibodies of PKC-ε, NM23-H1, UKHC, and GAPDH were used at dilutions of 1/5000, 1/1000, 1/200, and 1/1000, respectively for Western bolting.

## Results

### Morphology of differentiated cells

**Fig 1:**
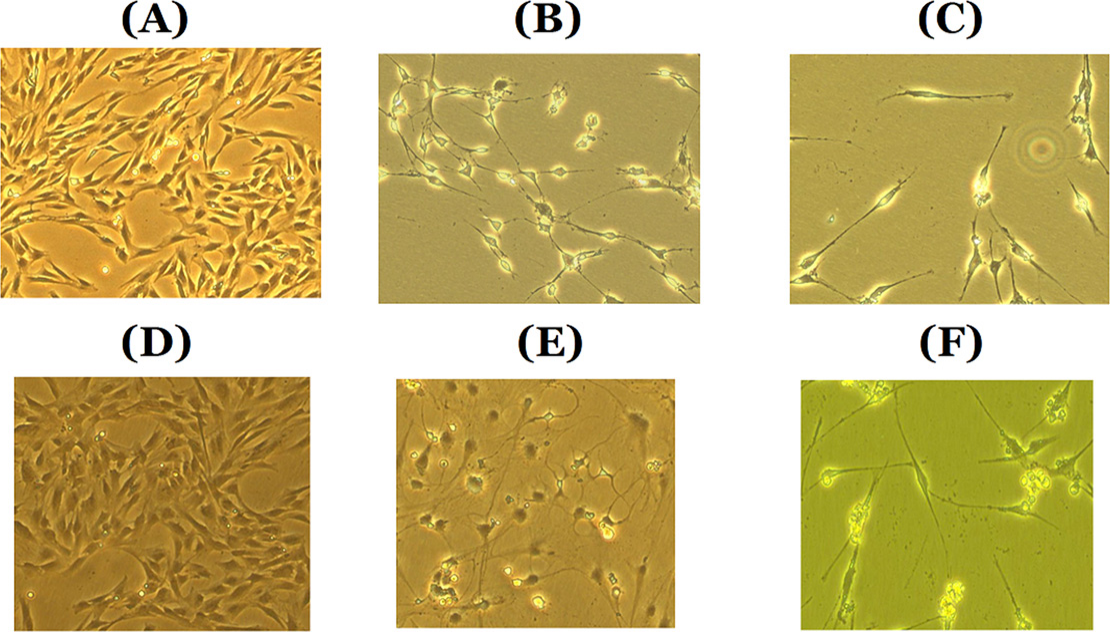
Cell morphology of *me*_SWH-treated MSCs (A, B, C in upper panel) and intact MSCs (D, E, F in lower panel) before and after neuronal differentiation induction. A and D are cells before induction; B and E are cells inducted with NIM for 5 and 7 days, respectively. C and F are cells induced with ERK inhibitor-containing NIM for 5 and 7 days, respectively.

Before induction, cells were spindle-shaped, which is the typical morphology of MSCs (Fig 1A and 1B). After neural differentiation, both non-treated and *me*_SWH-treated MSCs acquired a neural-like shape (Fig 1C and 1D). However, if the NIM supplemented with ERK inhibitor was used, neuronal differentiation was somewhat retarded. After the induction of MSCs for 7 days and *me*_SWH-treated MSCs for 5 days, both groups expressed the dopaminergic neuron marker TH and mature neuronal marker MAP2. However, neither the neural progenitor marker Nestin nor the glial cell marker GFAP were expressed. The expression of the neural markers TH and MAP2 was significantly reduced when the ERK pathway was blocked during neuronal induction.

### Secretion of BDNF by differentiated cells

**Fig 2:**
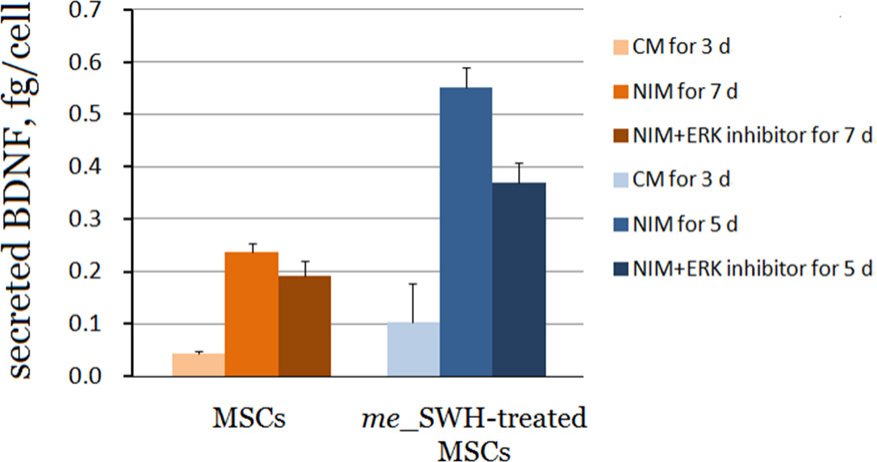
Amount of BDNF secreted by MSCs and *me*_SWH-treated MSCs. The intact MSCs were treated with NIM (with or without ERK inhibitor) for 7 days and *me*_SWH-treated MSCs for 5 days.Bar heights with SD error bars represent the average value of 3–7 replicate samples.

As shown in Fig 2, secreted level of BDNF in *me*_SWH-treated MSCs increased from 0.1 to 0.55 fg/cell after neural induction for 5 days. If the ERK inhibitor was present in the induction medium, the amount of BDNF was decreased to 0.33 fg/cell. Similar behavior was observed in the differentiation of intact MSCs. The secreted BDNF level increased from 0.045 to 0.23 fg/cell after induction for 7 days and decreased to 0.19 fg/cell when the ERK pathway was blocked. In summary, the differentiated cells secreted a significant level of BDNF for survival.

### Survival of differentiating cells

**Fig 3:**
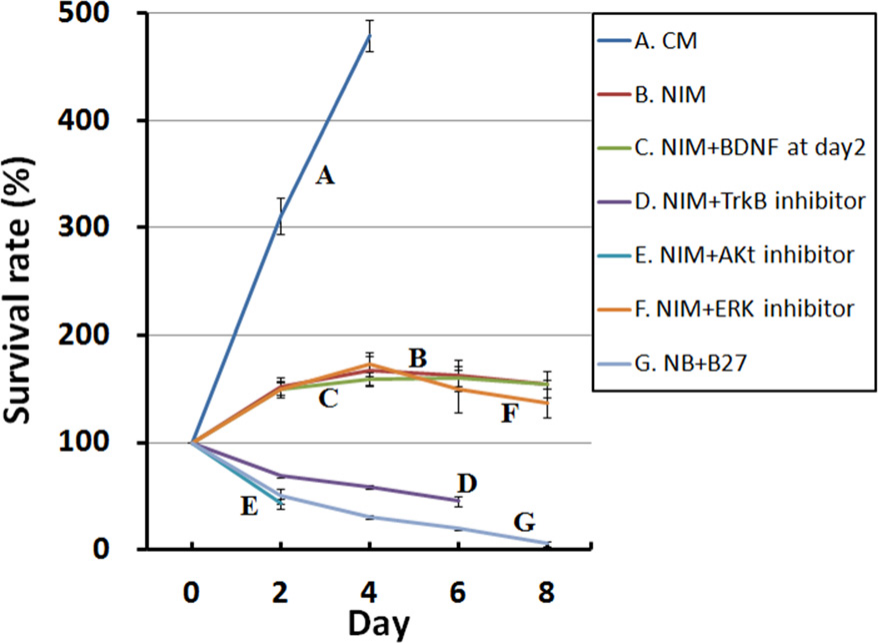
Survival rates for *me*_SWH-treated MSCs in different conditions. Each data point represents the average value of triplicate samples and each error bar represents the standard deviation.

The survival rate data indicate that *me*_SWH-treated MSCs showed a sustained proliferation in CM (Fig 3). After switching to NIM, cells maintained the characteristics of stem cells and continued to proliferate for 4 days. The cell number then decreased. When the cells were incubated in NIM supplemented with either TrkB or Akt inhibitor, the cell number decreased significantly after induction. In the presence of an inhibitor to the PI3K/Akt pathway, cell viability dropped significantly to 60% of its original value after 1 day of induction. However, the inhibition of the MAPK/ERK pathway resulted in a slight decrease in cell viability and BDNF secretion after 5 days. The addition of BDNF to NIM on day 2 reduced cell death to some extent. In the NIM, MSCs ceased to grow 2 days after the induction of neuronal differentiation. When either TrkB inhibitor or Akt inhibitor were present in the NIM, cells underwent apoptosis.

### Time courses of BDNF and signaling proteins

**Fig 4:**
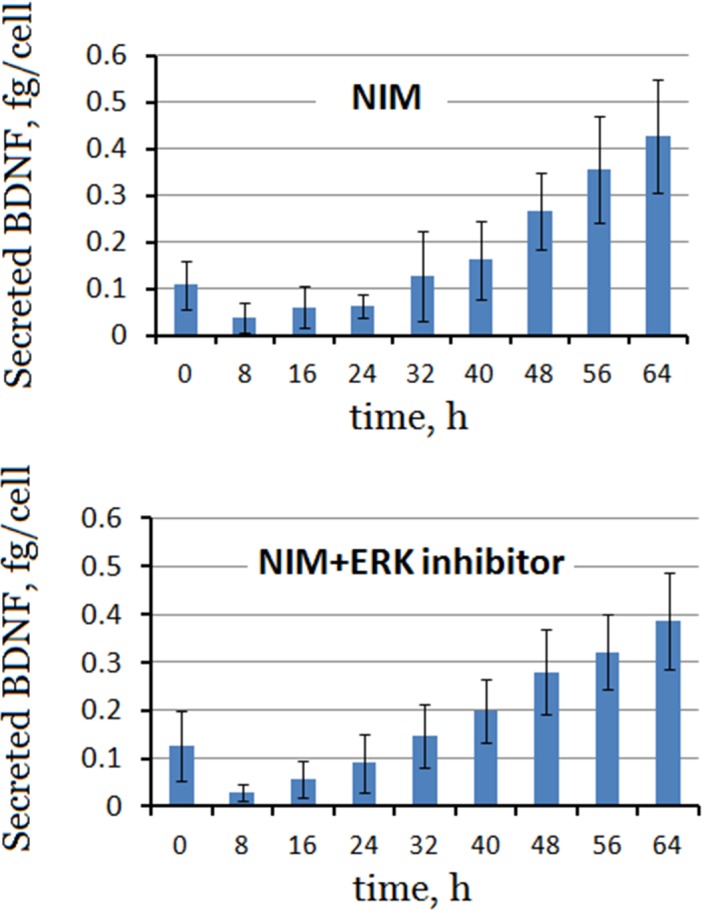
Time courses of BDNF secreted by differentiating *me*_SWH-treated MSCs in NIM in the absence (upper panel) and presence of ERK inhibitor (lower panel). Bar heights with SD error bars represent the average value of triplicate samples.

Fig 4 shows the time courses of BDNF secreted by cells in the NIM with and without ERK inhibitor. The level of BDNF secreted by MSCs decreased significantly immediately after induction. However, the differentiating MSCs began to secret BDNF at 24 h post-induction. The inhibition of the MAPK/ERK pathway had no significant effect on the secretion of BDNF.

**Fig 5:**
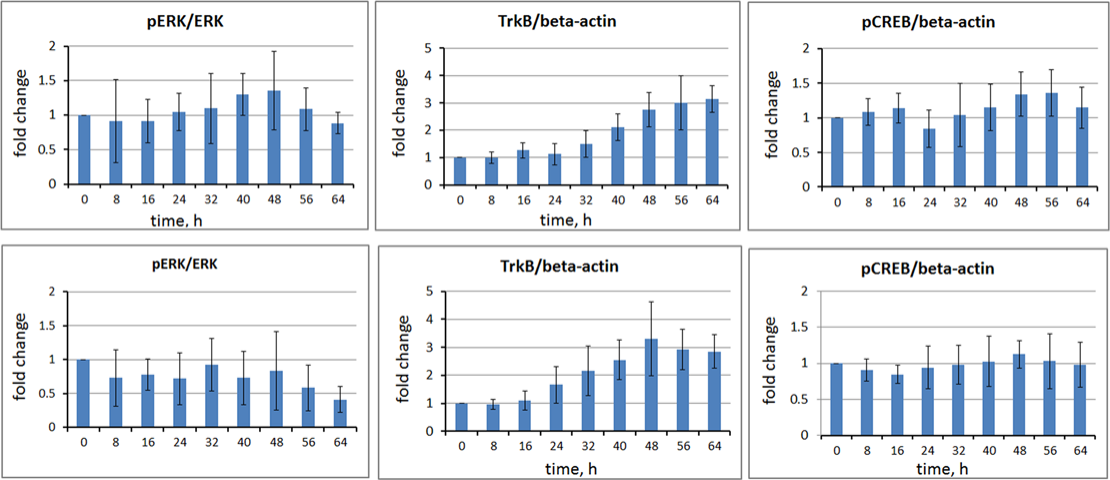
Time courses of relative expression levels of phosphorylated ERK, TrkB, and phosphorylated CREB for *me_*SWH-treated MSCs in NIM (upper panel) and in NIM with ERK inhibitor (lower panel). The relative levels of each protein at different time points were normalized to that at the time immediately prior to induction. The height of each bar in the graphs indicates the mean fold change. The error bars indicate the standard deviation of 3 or 4 replicate samples.

Fig 5 shows time courses of relative expression levels of phosphorylated ERK, TrkB and phosphorylated CREB for *me_*SWH-treated MSCs in NIM with and without ERK inhibitor. As shown in Fig 5, the level of phosphorylated ERK had a rebound increase 24 h after induction. However, in the presence of ERK inhibitor, the related level of phosphorylated ERK decreased gradually with time. Similarly, the phosphorylated CREB level increased initially with time and then decreased at 24 h. The phosphorylated CREB level then increased again to approximately 1.2 times the initial value at 56 h. An oscillation behavior was observed in the level of pCREB during the early stage of differentiation. This oscillation was smoothed out if ERK inhibitor was present in the NIM. The increase in TrkB levels was significant after 32 h of induction and increased to approximately 3.1 times the initial value at 64 h. When the ERK inhibitor was present in the induction medium, the TrkB level increased at 24 h and reached the highest value (approx. 3.3 times of the initial value) at 48 h. The TrkB level then decreased slightly over time.

### Differential expression of protein after neuronal differentiation

**Fig 6:**
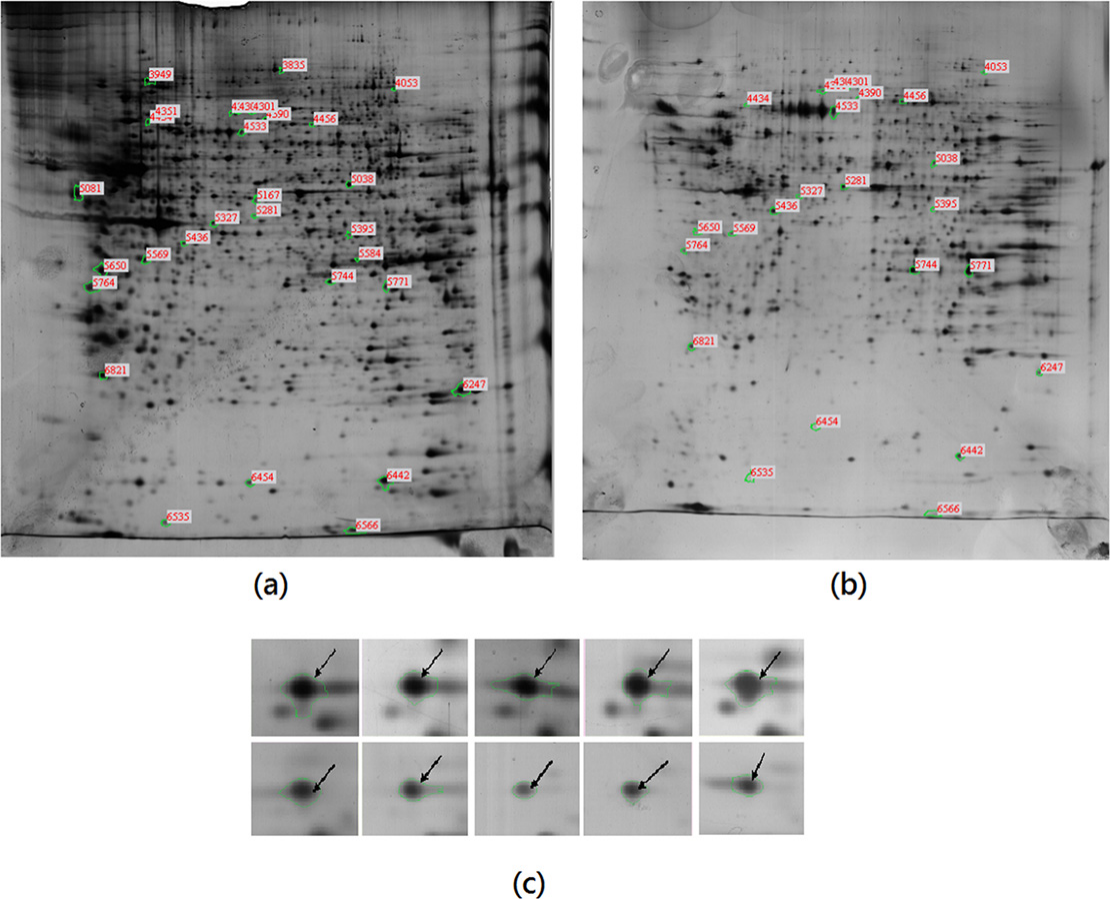
Two-dimensional gel electrophoresis of *me_*SWH-treated MSCs before (a) and after induction for neuronal differentiation for 5 days (b). The spot images of nucleoside diphosphate kinase A (NM23-H1) in different gels from *me_*SWH-treated MSCs before (upper panel) and after induction (lower panel) are also shown.

The 2-DE results indicate a significant change in the protein expression pattern (Fig 6). According to the t-test statistical analysis, 108 spots were differentially expressed with p < 0.05 as the threshold. Among these, 32 spots were up-regulated and 76 spots were down-regulated, with pre-induction cells being used as the control. When the p-value threshold was set to p < 0.01 and differential expression greater than 2 fold was taken into account, 30 spots were found to be differentially expressed, with 10 being up-regulated and 20 being down-regulated. These 30 protein spots were subjected to identification using the mass spectral method with software against NCBI or Swiss-Prot databases. Only three proteins were successfully identified and these are listed in Table 1. Among these three proteins, nucleoside diphosphate kinase A (NM23-H1) was down-regulated by the neuronal differentiation, whereas kinesin-1 heavy chain (KIF5B, UKHC) and protein kinase C epsilon type (PKC-ε) were up-regulated.

**Table 1:** Differentially expressed proteins in *me_*SWH-treated MSCs after neuronal differentiation.

Spot no.	Name of protein	Mean of %vol			p-value	
		Control	Experiment	Exp/Ctl		
4301	Protein kinase C epsilon type (PKC-ε)	0.0344	0.1411	4.099	0.0002	up-regulated
4307	kinesin-1 heavy chain (KIF5B)	0.0125	0.0937	7.467	0.0024	up-regulated
6442	Nucleoside diphosphate kinase A (NM23-H1)	0.4124	0.1009	0.245	0.0012	down-regulated

### Confirmation of differentially expressed proteins by western blot analysis

**Fig 7:**
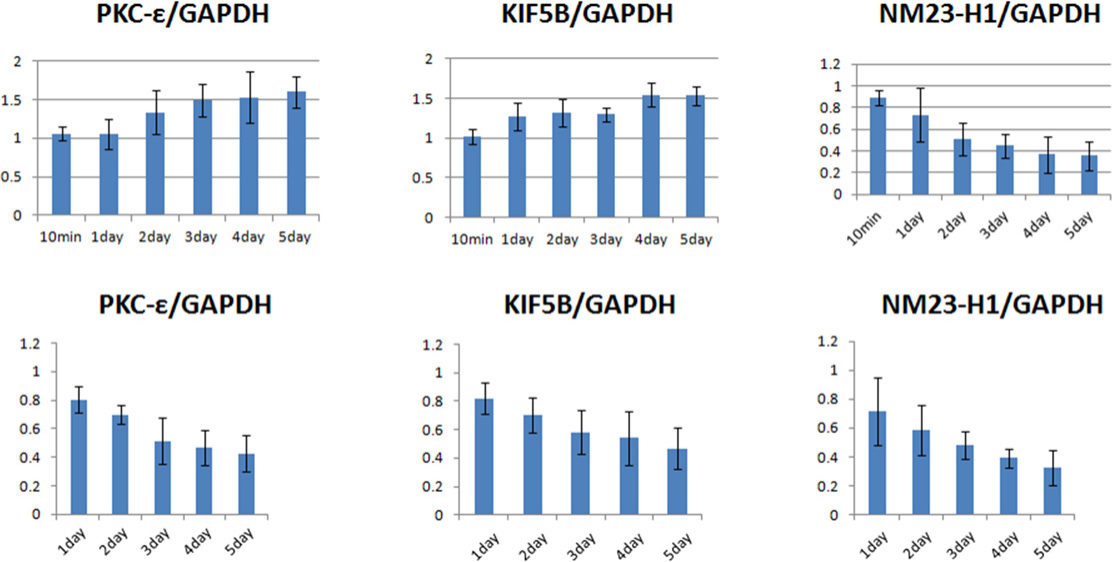
Time courses of relative expression levels of phosphorylated PKC-ε, TrkB, and NM23-H1 for *me_*SWH-treated MSCs in NIM (upper panel) and in NIM without SHH, bFGF and FGF-8 (lower panel).

Fig 7 shows the changes in cellular protein levels with time during neuronal differentiation. The western blotting results indicate that both PKC-ε and KIF5B increased with time of induction. Compared to the undifferentiated cells, the expression levels of PKC-ε and KIF5B were increased 154.7% and 159.6%, respectively, by the induction of neuronal differentiation for 5 days. However, after 5 days, the level of Nm23-H1 was reduced to 35.7% of its original level. According to the literature, KIF5B interacting to axonal synaptic vesicles is evident [21] and PKC-ε participates in several major signal pathways including the one results from SHH stimulation [22].

## Discussion

MSCs modified with targeting DNA hypermethylation of genes in the SWH pathway (*me*_SWH-treated MSCs) had better proliferation and differentiation ability than unmodified MSCs. After induction in NIM, both non-treated and *me*_SWH-treated MSCs showed a neural-like morphology and were TH positive. Their neural differentiation patterns were very similar to each other. However, *me*_SWH-treated MSCs differentiated faster than non-treated MSCs. After induction, the *me*_SWH-treated MSCs also secreted more BDNF (Fig 2). *me*_SWH-treated MSCs have demonstrated enhanced proliferation and differentiation and were not found to be tumorigenic in a nude mice assay [17]. Thus, *me*_SWH-treated MSCs are an ideal model for the study of stem cell differentiation.

**Fig 8:**
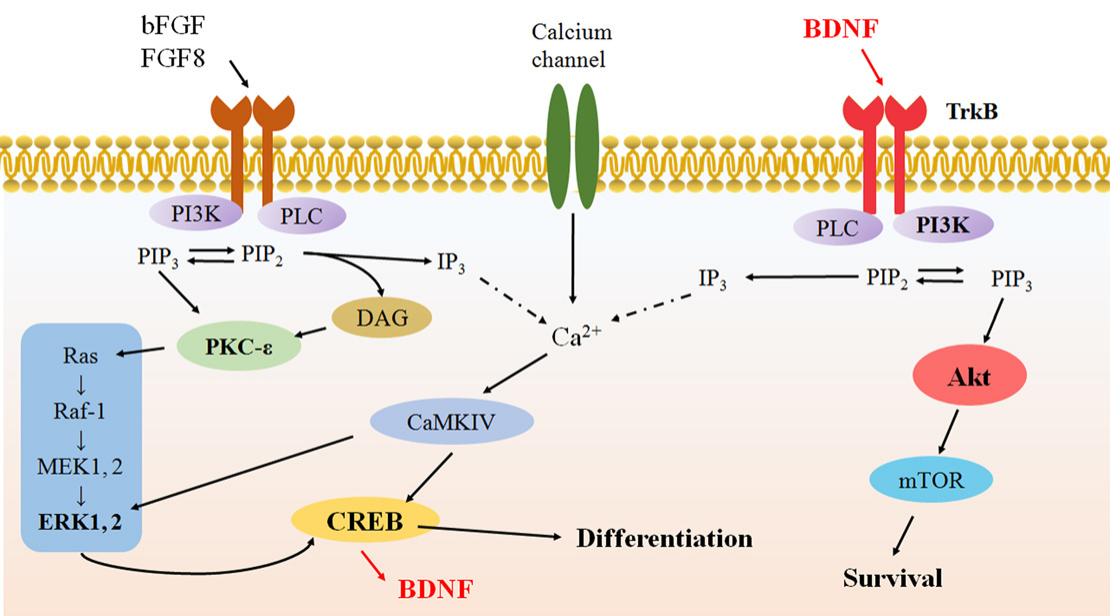
Schematic diagram showing the signaling pathways that support neuronal differentiation and survival.

The results from the cell viability assay (Fig 3) indicate that when the *me*_SWH-treated MSCs were cultured in CM a continuous increase in cell number can be observed because proliferation is one of the characteristics of stem cells. In the early stages of neural induction in NIM, although the increase in the number of cells was less than that in CM, some cells underwent cell division because of the stimulation by growth factors SHH, bFGF, and FGF8. However, the increase in cell number slowed down after 2 days of induction, suggesting that cells cease to grow before entering the differentiation phase. The peak number of cells was observed on the fourth day, and most cells were found to be neuron-like at this time. It is obvious that the PI3K/Akt pathway plays a key role in cell survival. When the Akt inhibitor was present in the NIM medium, the cells were unable to sustain growth and entered apoptosis, which is consistent with previous findings [23–24]. The secretion of BDNF by the differentiating cells in NIM (shown in Fig 2) allowed cells to maintain their survival level. However, if the BDNF receptor tyrosine receptor kinase B (TrkB) inhibitor k-252a was also present in the NIM, the inhibition of BDNF binding gradually led to cell apoptosis. The PI3K–Akt signaling pathway has been found to be a major pathway mediating neuronal survival [25]. According to the literature, the PI3K/Akt signaling pathway plays a critical role in mediating survival signals in a wide range of neuronal cell types [25]. Binding of BDNF to TrkB leads to the activation of Akt and eventually to the survival of neuronal cells. The increase in TrkB expression level with time suggested an uptake of the secreted BDNF by the differentiating cells for the suppression of apoptosis. Further, the binding of BDNF to TrkB activates CREB transcriptional pathways that collaborate with Akt-dependent pathways to support neuronal survival [26]. These pathways are outlined in Fig 8.

ERK is the up-stream kinase of CREB. If the ERK inhibitor (PD98059) was present in the NIM, the inhibition of MAPK/ERK pathway had only a minimal effect on the secretion of BDNF in the first 64 h (Fig 4). However, in the presence of ERK inhibitor, the cells gradually underwent apoptosis after day 4 but the differentiated cells survived in the NIM without ERK inhibitor. This suggests that inhibiting ERK could also reduce the secretion of BDNF, which is necessary for cell survival.

The activation of the nuclear transcription factor CREB is believed to be essential for neurogenesis. In the differentiating cells, CREB was active after the binding of growth factors bFGF and FGF-8 in the induction medium onto cell surface receptors. Binding of these growth factors activates receptor tyrosine kinase (RTK), which is linked to the downstream MAP kinase signaling cascade (MAPK/ERK signal transduction pathway) and results in the activation of CREB [27–28]. It can also regulate the activation of CREB via the Ca^2+^/calmodulin-dependent protein kinases (CaMKIV) signaling. The phosphorylation of ERK is the key step in the MAPK/ERK pathway. Phosphorylated ERK activates the phosphorylation of CREB, which initiates CREB-dependent gene transcription. Although the kinetics of CREB phosphorylation by EGF was transient, bFGF induced a prolonged pattern of CREB phosphorylation [29]. Similar to the differentiation of the neuroendocrine cell line PC12, transcription of CRE-dependent genes is required for the activation of the canonical signaling cassette Raf→MEK→ERK pathway [30].

As shown in Fig 5, the level of phosphorylated ERK had a rebound increase after 24 h of induction, suggesting that a sustained expression of pERK was required for neuronal differentiation. This is correlated with the finding that sustained ERK activity is required for cell differentiation in PC12 cells [31–32] and the duration of ERK activation is critical for cell signaling decisions [33].

Fig 5 shows that the addition of ERK inhibitor to NIM did inhibit the phosphorylation of ERK. Without the inhibitor, the phosphorylated ERK reached its peak value at 48 h after induction. The phosphorylated CREB reached its first peak value at 16 h and then gradually decreased, followed by a gradual increase in a second peak at approximately 48–56 h. If the NIM supplemented with ERK inhibitor was used, the levels of phosphorylated ERK and CREB were slightly lower (Fig 5) and the neuronal differentiation was somewhat retarded. On the basis of the comparison of the time course of ERK and CREB phosphorylations, we can deduce that when cells were induced by growth factors bFGF and FGF8, the MAPK/ERK pathway was activated, resulting in the phosphorylation of CREB. Downstream of CREB phosphorylation causes the secretion of BDNF and its binding to TrkB, activating the MAPK/ERK pathway, which generates a second CREB phosphorylation peak. When ERK inhibitor was present, there was less CREB activation leading to a reduction in the secretion of BDNF.

Fig 5 also shows the relationship between the synthesis of TrkB receptor and secretion of BDNF over time. In the NIM conditions, the TrkB receptor increased with time until 56 h. BDNF binding presumably stimulated phosphorylation of ERK, resulting in phosphorylated ERK reaching a peak at the same time. At this time, most cells started to gain neural-like morphology. This indicates that differentiated cells overexpressed TrkB receptors and binding BDNF in order to promote their functional maturation and survival. These results confirmed that BDNF can facilitate the maturation of MSC-derived dopamine progenitors [34].

Proteins that were significantly differentially expressed as shown in 2-D gel electrophoresis included Nm23/Nucleoside diphosphate kinase A protein (NM23-H1), kinesin-1 heavy chain (KIF5B), and protein kinase C epsilon type (PKC-ε). These proteins could be closely related to neurogenesis. Results from western blotting confirmed that both PKC-ε and KIF5B increased with time of induction. KIF5B is found in nerve and glial cells, functions to maintain synaptic function and regulation of neural plasticity in mature neurons, and plays an important role in the presynaptic nerve function. Our results could match a previous finding that KIF5B is up-regulated in the developing rat CNS [35]. PKC-ε has been shown to have an anti-apoptotic effect in many cell types [36]. Our results indicated that the PKC-ε level measured by western blotting increased with time until 3 days after induction and then remained at a high level. The correlation between PKC-ε and TrkB suggested that there is cross-talk between PKC-ε and the PI3K/Akt signaling pathway, which plays a role in the survival of differentiated neurons.

## Conclusion

Due to their enhanced proliferation and differentiation ability, *me*_SWH-treated MSCs served as a good model for the study of signaling conduction in MSC differentiation. In this work, *me*_SWH-treated MSCs were induced to differentiate into neuronal cells *in vitro* using a neurobasal medium supplemented with B27, SHH, bFGF and FGF8. After 5 days of differentiation, the length of me_SWH hMSCs increased 1.52 times in comparison to undifferentiated cells. The results indicate that in the presence of an inhibitor of the phosphoinositide 3-OH kinase (PI3K)/Akt pathway, cell viability decreased significantly to 60% of its original value 1 day after induction. Two signaling pathways, the PI3K/Akt and MAPK/ERK pathways, could play important roles in the neuronal differentiation of MSCs. The PI3K/Akt pathway serves to maintain cell survival during neural differentiation; whereas the function of the MAPK/ERK pathway is probably to enhance the maturation of differentiated MSCs. Blocking of the PI3K/Akt pathway by the Akt inhibitor caused significant cell death. We found that the expression of TrkB correlated well with the secretion of BDNF during differentiation. The differentiated cells secreted BDNF to activate TrkB via the PI3K/Akt signaling pathway in order to maintain cell survival. During neuronal differentiation, sustained ERK activity was achieved by activation of the MAPK/ERK pathway due to the binding of growth factors bFGF and FGF in the early phase and then later by the binding of BDNF to TrkB. In addition, the conventional proteomic method was used to investigate the change in cellular protein expression as a result of neuronal differentiation. Three proteins—nucleoside diphosphate kinase A (NM23-H1), kinesin-1 heavy chain (KIF5B, UKHC), and protein kinase C epsilon type (PKC-ε)—were found to be differentially expressed by the neuronal differentiation. Via the secretion and binding of BDNF, there is cross-talk between PKC-ε and the PI3K/Akt signaling pathway, which is essential for the survival of differentiated neurons.

## Abbreviations

BDNF: brain-derived neurotrophic factor
CREB: cAMP response element-binding protein
CaMKIV: Ca^2+^/calmodulin-dependent protein kinase IV
FGF2, bFGF: fibroblast growth factor 2, basic fibroblast growth factor
FGF8: fibroblast growth factor 8
GAPDH: glyceraldehyde 3-phosphate dehydrogenase
KIF5B: UKHC: kinesin-1 heavy chain
MAP2: microtubule-associated protein 2
*me*_SWH-treated MSCs: MSCs modified with targeting DNA hypermethylation of genes in the Salvador/Warts/Hippo pathway
MAPK/ERK pathway: mitogen-activated protein kinase/extracellular signal-regulated kinase pathway
NM23-H1: nucleoside diphosphate kinase A
PI3K/Akt pathway: phosphoinositide 3-OH kinase/Akt pathway
PKC-ε: protein kinase C epsilon type
SWH pathway: Salvador/Warts/Hippo pathway
SHH: Sonic hedgehog
TH: tyrosine hydroxylase
TrkB: tyrosine receptor kinase B
